# The Roles of Buyang Huanwu Decoction in Anti-Inflammation, Antioxidation and Regulation of Lipid Metabolism in Rats with Myocardial Ischemia

**DOI:** 10.1093/ecam/neq028

**Published:** 2011-03-10

**Authors:** Yu Liu, Rong Lin, Xiaolian Shi, Zhiyuan Fang, Weirong Wang, Qinqin Lin, Jiye Zhang, Hui Zhang, Qiaoli Ji

**Affiliations:** ^1^Department of Pharmacology, Medical School of Xi'an Jiaotong University, Xi'an, Shaanxi 710068, China; ^2^Shaanxi Provincial People's Hospital, Xi'an, Shaanxi 710068, China

## Abstract

Buyang Huanwu Decoction (BYHWD) is a well-known Chinese medicine formula. Recent studies have reported that BYHWD can be used to treat ischemic heart disease. This study investigated the potential mechanism underlying the roles of BYHWD in alleviating the myocardial ischemia induced by isoproterenol (ISO) in rats. Different doses of BYHWD (25.68, 12.84 and 6.42 g kg^−1^) were lavaged to rats, respectively. Then the expression of the cluster of differentiation 40 (CD40) in the mononuclear cells was measured using flow cytometry, and the expressions of CD40 and its ligand (CD40L) in myocardial tissues were determined by western blotting. The serum biochemical values of superoxide dismutase (SOD) activity, the malondialdehyde (MDA) level and the free fatty acid (FFA) content were measured. The results showed that the SOD activities of BYHWD groups were significantly higher than that of the ISO group, while the MDA levels and FFA contents of all BYHWD groups were lower than that of the ISO group. BYHWD could decrease the expression of CD40 in the mononuclear cells and the CD40 and CD40L expressions in myocardial tissues. Our data suggest that the roles of BYHWD are not only related to its antioxidative action and regulation of lipid metabolisms, but also to the inhibition of inflammatory pathway by the decreased CD40 and CD40L expressions in rats with myocardial ischemia.

## 1. Introduction

Ischemic heart disease (IHD), one of the leading chronic disorders, is often highlighted in public health [1]. In recent years, inflammation has been recognized as a major driving force in ischemic process, and increasing evidence has shown that enhanced levels of inflammatory markers, including C-reactive protein, NF-kappa B and matrix metalloproteinases, are related to IHD [2–4]. During the past decade, the cluster of differentiation 40 (CD40) and its ligand (CD40L), as important inflammatory regulators, have been found to play a role in atherosclerosis [5]. Studies have revealed that CD40/CD40L plays an important role in IHD and that the inhibition of CD40/CD40L may be used as a new therapeutic strategy for IHD treatment [6].

Buyang Huanwu Decoction (BYHWD), a well-known Chinese medicine formula, has been extensively used for improving neurological functional recovery in stroke-induced disability in China for centuries. Few recent studies have reported that BYHWD can be used to treat ischemic coronary heart disease by relieving angina pectoris [7, 8]. Investigations have showed that BYHWD has the capacity to inhibit inflammatory pathway in rats with cerebral ischemia and in stroke patients [9, 10]. Nevertheless, little is known about the mechanism by which BYHWD alleviates IHD. In the previous study, we have found that BYHWD cannot only improve the electrocardiogram of rats with myocardial ischemia, but also decrease the activities of lactate dehydrogenase, creatine kinase and aspartate aminotransferase, which are related to the inhibition of isoproterenol (ISO)-induced nitric oxide synthase in rats [11]. ISO induces alterations in membrane permeability, resulting in the loss of function and integrity of myocardial membranes at dosages exceeding the physiological concentrations [12]. To further research the potential mechanism of BYHWD, the present study examined the expression of CD40 in the mononuclear cells, the expressions of CD40 and CD40L in myocardial tissues, the superoxide dismutase (SOD) activity, malondialdehyde (MDA) level and the content of free fatty acid (FFA) in the serum to assess the roles of the BYHWD in anti-inflammation, antioxidation and regulation of lipid metabolisms in the myocardial ischemic rats.

## 2. Methods

### 2.1. Materials

Trihydroxymethyl aminomethane (Tris), glycine, sodium dodecyl sulfate (SDS), acrylamide and bis-acrylamide were purchased from Amresco (Amresco, USA). Anti-CD40, anti-CD40L antibody and anti-*β*-actin antibody were obtained from Santa Cruz Biotechnology (Santa Cruz, CA, USA). Goat anti-rabbit horseradish peroxidase-linked antibody, BCA Protein Assay kit, Mem-PER Eukaryotic Membrane Protein Extraction Reagent Kit, BlueRanger Prestained Protein Molecular Weight Marker Mix and SuperSignal West Pico Chemiluminescent Substrate were obtained from Pierce Chemical Company (Pierce, USA). Goat anti-rabbit FITC-conjugated IgG antibody was purchased from Jackson immunoresearch (Jackson, USA). SOD, FFA and MDA assay kits were produced by the Institute of Nanjing Jiancheng Biology Engineering (Nanjing, P. R. China). Protein Extraction Reagent (RIPA) kit was purchased from Bioteke Corporation (Beijing, P. R. China). ISO was purchased from Sigma Biotechnology (Sigma, USA). Compound Danshen Dropping Pills (CDDP) was obtained from Tianjin Tianshili Pharmaceuticals Company (Tianjin, P. R. China). All other agents used in the study were of the commercially available grade.

### 2.2. Preparation of BYHWD

BYHWD was made from seven medicinal components: 120 g milkvetch root, 6 g Chinese angelica, 3 g Szechwan lovage rhizome, 4.5 g red peony root, 3 g earth worm, 3 g peach seed and 3 g safflower. The seven crude drugs were purchased from Xi'an pharmacy of Beijing Tongrentang and authenticated by experts in pharmacognosy. The mixture of the crude drugs was soaked in distilled water and boiled twice [13]. The resulted BYHWD was filtered through a mesh, and the filtrate was concentrated to 3 g mL^−1^ by a vacuum pump and stored at 4°C until use. CDDP was used here as a positive control drug. The major ingredient of CDDP is Danshen. Danshen is an important traditional Chinese herbal medicine that has extensive clinical applications for cardiovascular diseases, including acute myocardial infarction [14].

### 2.3. Experimental Animals and Model

The experiments were performed on 60 male adult Sprague-Dawley (SD) rats, each weighing 180–200 g, provided by Medical Experimental Animal Center of the Xi'an Jiaotong University (Xi'an, China). Animals were randomly allocated to six equal number groups: control, ISO, pre-treatment with CDDP (0.073 g kg^−1^) and three pre-treatment with BYHWD (25.68, 12.84 and 6.42 g kg^−1^, resp.). ISO-induced myocardial ischemic animal model was established by intraperitoneal injection of ISO (0.03 g kg^−1^) once a day for 3 days [15, 16]. Under anesthesia condition, if electrocardiogram recording indicated a significant ST segment fall of >0.l mV, it was considered that this myocardial ischemia model has been established, as reported previously [11]. It is clinically important if ST segment elevated or fell as it can be a sign of ischemia and hyperkalemia.

Rats in BYHWD and CDDP groups were intragastrically administered with BYHWD and CDDP, respectively, once a day for 14 days. From day 12, ISO was injected to all the animals except those in the control group. By the end of day 14, rats were anesthetized with pentobarbital sodium, and heart tissue and blood samples were collected for further examinations. The experimental protocol was in accordance with the National Institutes of Health Guide for Care and Use of Laboratory Animals and was approved by the Institutional Animal Care Committee of Xi'an Jiaotong University.

### 2.4. CD40 Expression in Mononuclear Cells

The expression of CD40 in the mononuclear cells was measured using flow cytometry, as previously described [17]. Briefly, heparin was added into the collected blood, and then the heparin-anticoagulated blood was incubated with the rabbit anti-rat CD40 polyclone antibody for 30 min at room temperature (RT), washed twice with phosphate buffered saline (PBS, pH 7.4) and centrifuged at 1000 g for 3 min before incubating with the goat anti-rabbit FITC-conjugated IgG for 30 min at RT. Finally, the blood so derived was immediately fixed in 4% paraformaldehyde PBS and analyzed for the determination of the CD40 expression in mononuclear cells using a Becton Dickinson FACScan flow cytometry (BD Biosciences, USA).

### 2.5. CD40 and CD40L Expressions in Myocardial Tissues

To investigate the potential mechanisms by which BYHYD protects myocardium from ischemia, the expressions of CD40 and CD40L in myocardial tissues were determined by western blotting. Membrane protein of myocardial tissues was extracted following the instructions of the Mem-PER Eukaryotic Membrane Protein Extraction Reagent Kit. Protein concentration was quantified using a BCA protein quantity assay kit before 12% sodium dodecyl sulfate polyacrylamide gel electrophoresis (SDS-PAGE) under 4°C. Then the protein was transferred onto microporous polyvinylidene fluoride membranes in running buffer containing 20% methanol. After non-specific sites were blocked with 5% milk-Tris buffered saline-Tween 20 (TBST), the membranes were incubated with anti-CD40 antibody, anti-CD40L antibody and anti-*β*-actin antibody overnight, respectively. Then, a horseradish peroxidase-linked antibody was employed as a secondary antibody after the membranes were washed in TBST. The bands of interest were detected using an enhanced chemiluminescent technique. Densities of the bands were measured using an IBAS ReL2.0 image analyzer (Option Company, Belgium).

### 2.6. SOD Activity and MDA Level in Serum

SOD activity and MDA level in the serum were measured as previously described [18]. In brief, when rats were sacrificed, blood was collected and centrifuged at 800 g for 5 min; then the serum was collected for the detection of SOD activity and MDA level by xanthine oxidase and thiobarbituric acid methods, respectively. The assay kits were of the commercially available grade, and all the operations were carried out following the instructions of the kit. The findings were detected using an ultraviolet/visible scanning spectrophotometer (Beckman Coulter, Inc. USA).

### 2.7. FFA Content in Serum

FFA content in the serum was determined by colorimetric method after extraction. The assay kit was of the commercially available grade, and all the operations were carried out following the instructions of the kit. The findings were detected using an ultraviolet/visible scanning spectrophotometer (Beckman Coulter, Inc. USA).

### 2.8. Statistical Analysis

Data were expressed as mean ± standard deviation (SD). The difference between ISO and control groups was tested by Student's *t*-test, and comparisons between all the treated groups and ISO group were performed using one-way analysis of variance (ANOVA) with a Dunnetts' test. Two-tailed values of *P* < .05 were considered to indicate statistical significance. All data were analyzed by using SPSS 11.0 software.

## 3. Results

### 3.1. CD40 Expression in Mononuclear Cells

**Figure 1:**
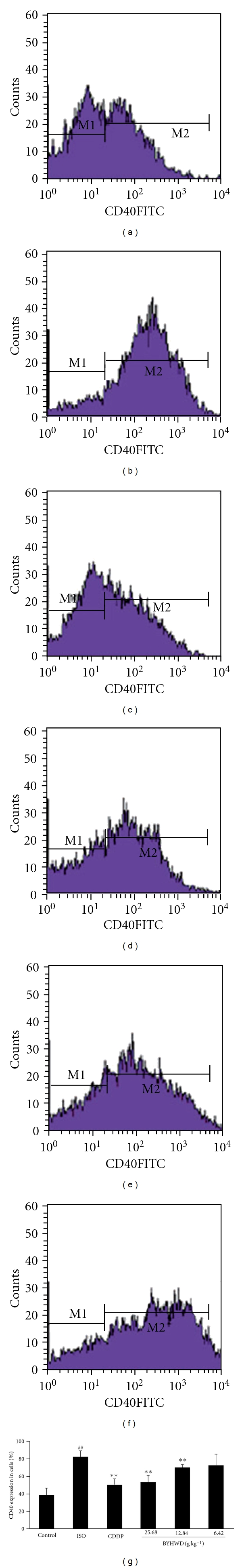
CD40 expression in mononuclear cells (scale bar = 100 *μ*m). A myocardial ischemic animal model was established by administering ISO to SD rats. BYHWD was administered in different doses (25.68, 12.84 and 6.42 g kg^−1^) to this model. The expressions of CD40 in mononuclear cells in different groups were detected by flow cytometry. Results were expressed as mean ± SD. ^*##*^ *P* < .01, compared with the control group. **P < .01, compared with the ISO group. (a) Control group. (b) ISO group. (c) CDDP group (0.073 g kg^−1^). (d) BYHWD group (25.68 g kg^−1^). (e) BYHWD group (12.84 g kg^−1^). (f) BYHWD group (6.42 g kg^−1^).

The cell-surface expression of CD40 in the rat mononuclear cells was determined using flow cytometry. As is shown in Figure 1, the percentage of positive cells in the ISO group increased significantly, compared with that of the control group (*P* < .01). Pre-treatment with BYHWD or CDDP resulted in a decreased percentage of positive cells. There was a statistical difference between the ISO group and the CDDP group and 12.84 and 25.68 g kg^−1^ BYHWD groups (*P* < .01).

### 3.2. CD40 and CD40L Expressions in Tissues

**Figure 2:**
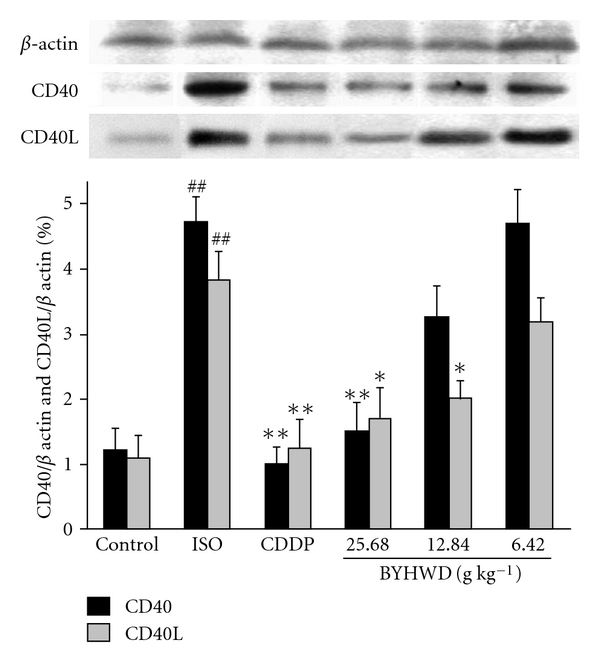
CD40 and CD40L expressions in tissues (scale bar = 100 *μ*m). A myocardial ischemic animal model was established by administering ISO to SD rats and BYHWD was administered in different doses (25.68, 12.84 and 6.42 g kg^−1^) to this model. Bar graph (below) showed the CD40 and CD40L densities analyzed by computer-assisted image quantification. Results were expressed as mean ± SD. ^*##*^ *P* < .01, compared with the control group. *P < .05, **P < .01 compared with the ISO group. The films (upper) showed the expressions of CD40 and CD40L in myocardial tissues of different groups detected by western blotting.

As is shown in Figure 2, the ISO group presented a high level of CD40 and CD40L expressions, compared with the control group (*P* < .01). Pre-treatment with BYHWD (25.68 g kg^−1^) or CDDP showed decreased expressions of CD40 and CD40L. The ISO group showed a statistical difference in CD40L expression (12.84 and 25.68 g kg^−1^ BYHWD groups, *P* < .05; CDDP group, *P* < .01), and a significant difference in CD40 expression (25.68 g kg^−1^ BYHWD group and CDDP group, *P* < .01) from the BYHWD and CDDP groups.

### 3.3. Effect on SOD and MDA in Serum

The results (Table 1) indicated that the ISO group showed a significantly decreased SOD activity (*P* < .01) and an increased MDA level (*P* < .01) compared with the control group. Pre-treatment with BYHWD (6.42, 12.84 and 25.68 g kg^−1^) or CDDP increased the SOD activity, and the SOD activities of BYHWD (6.42, 12.84 and 25.68 g kg^−1^) groups and CDDP group were markedly higher than that of the ISO group (*P* < .05, *P* < .01). On the other hand, BYHWD decreased the MDA level, and the MDA levels of the three BYHWD groups (6.42 g kg^−1^, *P* < .05; 12.84 and 25.68 g kg^−1^, *P* < .01) and CDDP group (*P* < .01) were markedly lower than that of the ISO group.

### 3.4. Effect on FFA in Serum

**Figure 3:**
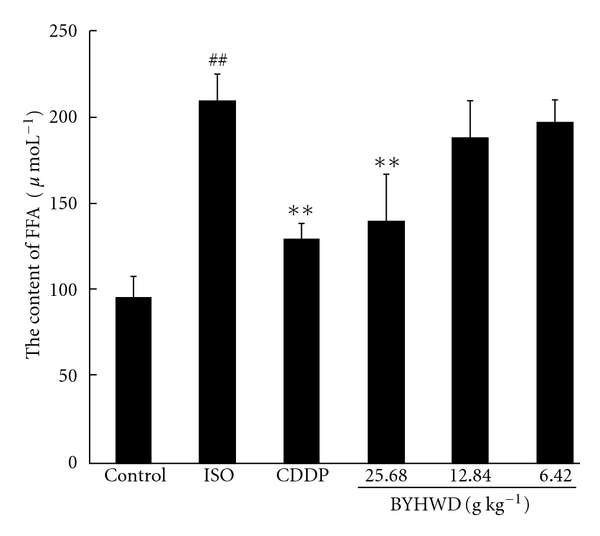
Effect on FFA in serum (scale bar = 100 *μ*m). A myocardial ischemic animal model was established by administering ISO to SD rats and BYHWD was administered in different doses (25.68, 12.84 and 6.42 g kg^−1^) to this model. The content of FFA was detected using colorimetric method. Results were expressed as mean ± SD. ^*##*^ *P* < .01, compared with the control group. **P < .01, compared with the ISO group.

As shown in Figure 3, the content of FFA in the ISO group increased significantly (*P* < .01), compared with that of the control group. Pre-treatment with BYHWD decreased the serum content of FFA. The ISO group showed a statistical difference in the FFA content from the BYHWD groups (25.68 g kg^−1^, *P* < .01) and the CDDP group (*P* < .01).

## 4. Discussion

It is now well accepted that IHD is a multifactorial disease caused calcium overload, oxidative stress, inflammation, and so forth. It has been found that CD40/CD40L signaling pathway, as an important inflammatory regulative pathway, plays an important role in the process of IHD [19]. CD40 and CD40L are molecules with prothrombotic and proinflammatory properties, expressed in a variety of tissues such as immunity cells, vascular wall, and above all, platelets. CD40L-CD40 interaction may induce inflammatory responses that lead to atherosclerosis, plaque destabilization and thrombosis [20]. In the present study, we found that the ISO-induced myocardial ischemic rats presented a high level of CD40 and CD40L expressions, compared with the control group, indicating that myocardial ischemia is related to CD40/CD40L inflammatory pathway. In addition, other reports have shown that increased co-expression of CD40/CD40L has been observed in patients with acute coronary syndrome [21–23], stable coronary artery disease [24, 25] and acute myocardial infarction [26, 27]. Reversing of the over-expression of CD40 and/or CD40L by simvastatin and losartan can improve cardiovascular injury [28]. Experimental studies have also shown that the inhibition of CD40/CD40L pathway not only decreases vascular inflammation and prevents the development of atherosclerosis [29], but also protects ischemic coronary artery in rabbits [6]. Increasing evidence has indicated that inhibition of CD40/CD40L pathway may be a new therapeutic strategy for treating IHD. However, Remskar et al. [30] have found that CD40 signaling plays a significant role in the inhibitory effect of T lymphocytes on intimal thickening after arterial injury, but the mechanism for the increased intimal thickening in CD40L−/− mice is unclear. In addition, Andreas Zirlik et al. [31] have demonstrated that CD40 ligand mediates inflammation independently of CD40 by interaction with Mac-1. Their study has made the surprising finding that CD40 does not contribute to CD40L-induced atherogenesis. These contradictions indicate that many complex problems need to be solved in the future.

BYHWD is a well-known traditional Chinese medicine formula made up of seven kinds of Chinese medicine. In clinic, BYHWD has been widely used for therapy of ischemic cerebral disease by activating blood circulation and improving neurological functional recovery [32, 33]. Recently, clinical studies have found that BYHWD can be used as a therapy for IHD by relieving angina pectoris, improving myocardial ischemia and decreasing the volume of myocardial infarction [34–36]. In the previous study, we have found that BYHWD can significantly inhibit the expression of CD40L in myocardial tissues by immunohistochemistry [11]. To further affirm the effect of BYHWD on the expression of CD40/CD40L system, we measured the expression of CD40 in the mononuclear cells using flow cytometry and the expressions of CD40 and CD40L in myocardial tissues by western blotting. In accordance with the previous results, we found that BYHWD (25.68 and 12.84 g kg^−1^) reduced the expression of CD40 in the mononuclear cells and inhibited the expressions of CD40 and CD40L in myocardial tissues, which suggests that the roles of BYHWD on myocardial ischemia may be related to the inhibition of CD40 and CD40L expressions. In the previous studies, we have reported that Tanshinone IIA can suppress the inflammation in atherosclerotic lesion by decreasing the CD40 expression [18, 37]. Data from the present study suggest that inhibition of inflammatory pathway by decreasing the CD40/CD40L expression may be one of the potential mechanisms by which BYHWD alleviates IHD. In addition, it has been found that BYHWD can inhibit the protein and mRNA expressions of intercellular adhesion molecule-1 and vascular cell adhesion molecule-1, and restrain inducible nitric oxide synthase in rats with blood stasis [10, 29, 38]. BYHWD can also inhibit caspase-1 expression and decrease the production of inflammatory cytokine in middle cerebral artery occlusion rats [39]. All these findings indicate that the roles of BYHWD are related to the inhibition of inflammation.

As we have known, oxidation plays an important role in diseases such as atherosclerosis, ischemia/reperfusion injury and inflammation. A large number of oxidative agents can increase the release of inflammatory factors such as TNF-*α*, IL-8 and NO [40]. Antioxidative therapy can inhibit the production of these inflammatory factors [41, 42]. It has reported that both inflammatory markers and oxidation products increase significantly in coronary heart disease and that there is a positive relationship between oxidation and coronary heart disease [43]. SOD is a major antioxidative factor whose activity reflects the antioxidative status in the body, while MDA is a major oxidative product whose level reflects the oxidative stress status in the body. Many traditional decoctions are recognized as sources of natural antioxidants that can protect biological system [44]. In the present study, we found that BYHWD increased the SOD activity and decreased the MDA level in the serum of the rat model with ISO-induced myocardial ischemia, which suggests that the roles of BYHWD on myocardial ischemia is, to some extent, related to its antioxidative action.

FFA is a main energy source of myocardium. When myocardial ischemia occurs, the energy metabolism is disturbed and FFA is accumulated abundantly in the myocardial tissues and blood [45–47]. In the present study, our results shows that the content of FFA in the myocardial ischemic rats increased significantly, compared with that of the normal rats, and that BYHWD reduced the FFA content in the serum of myocardial ischemic rats. Previous studies have indicated that high concentration of FFA can lead to myocardium injury, dysfunction of the endothelium and atherosclerosis [48, 49]. Moreover, it has been reported that high concentration of FFA is involved in the local inflammation in myocardial ischemia [50], and that high concentration of FFA is an independent risk factor for cardiac sudden death in acute coronary disease [51]. Rectifying the impaired metabolism of fatty acid may effectively relieve myocardial ischemia [52]. All these studies suggest that BYHWD has a protective effect on myocardial ischemia by rectifying impaired metabolism of FFA in rats.

**Figure 4:**
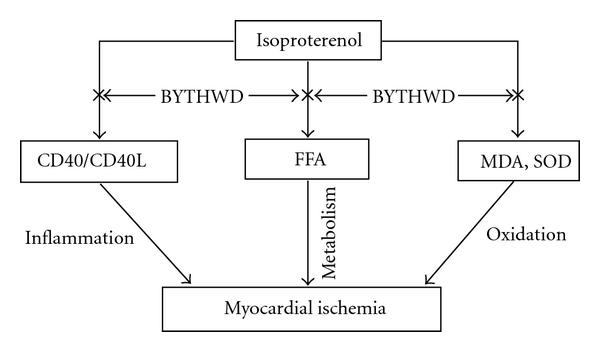
The results are summarized in the diagram. The roles of BYHWD are not only related to its antioxidative action and regulation of lipid metabolisms, but also to the inhibition of inflammatory pathway by the decreased CD40 and CD40L expressions in rats with myocardial ischemia.

In conclusion, the present study shows that BYHWD has a protective effect against ISO-induced myocardial ischemia, and this effect may be attributed to the down-regulation of CD40 and CD40L expressions. Moreover, the roles of BYHWD are related to the antioxidative activity and regulation of lipid metabolisms. These results are summarized in Figure 4. Our results provide an explanation for the relationship between the anti-inflammation and the protective effect of BYHWD on myocardial ischemia. The findings of the present study may shed light on the pharmacological basis for the clinical application of the traditional Chinese medicine in the treatment of ischemic cardiovascular diseases.

## Figures and Tables

**Table 1:** Effect on SOD and MDA in serum.

Group	Dose (g kg^−1^)	SOD (U mL^−1^)	MDA (nmol mL^−1^)
Control	—	389.97 ± 17.45	6.37 ± 2.49
ISO	—	233.19 ± 26.77*	19.25 ± 3.78*
CDDP	0.073	314.52 ± 21.59**	11.06 ± 6.19**
BYHWD	25.68	337.08 ± 29.85**	11.73 ± 4.16**
BYHWD	12.84	334.01 ± 18.74**	13.66 ± 2.21**
BYHWD	6.42	308.75 ± 33.71***	15.15 ± 3.94***

All the data are shown as mean ± SD, *n* = 10. *P < .01 compared with the control group; **P < .01 and ***P < .05 compared with the ISO group.
